# Whole genome resequencing identifies the *CPQ* gene as a determinant of ascites syndrome in broilers

**DOI:** 10.1371/journal.pone.0189544

**Published:** 2018-01-02

**Authors:** Shatovisha Dey, Alia Parveen, Katy J. Tarrant, Timothy Licknack, Byungwhi C. Kong, Nicholas B. Anthony, Douglas D. Rhoads

**Affiliations:** 1 Program in Cell and Molecular Biology, University of Arkansas, Fayetteville, Arkansas, United States of America; 2 Department of Biological Sciences, University of Arkansas, Fayetteville, Arkansas, United States of America; 3 Department of Animal Sciences and Agricultural Education, California State University, Fresno, California, United States of America; 4 Department of Poultry Sciences, University of Arkansas, Fayetteville, Arkansas, United States of America; Xiamen University, CHINA

## Abstract

**Background:**

Ascites syndrome is the most severe manifestation of pulmonary hypertension in fast-growing broilers. The disease can be attributed to increased body weights of birds, where the higher metabolic load is not matched by sufficient oxygen supply to the cells and tissues. Although there are environmental components, the disease exhibits moderate to high heritability. The current study uses high throughput whole genome resequencing (WGR) to identify genes and chromosomal regions associated with ascites.

**Results:**

The WGR data identified the *CPQ* gene on chromosome 2. The association was confirmed by genotyping a large collection of DNAs from phenotyped birds from three distinct broiler lines using SNPs in intron 6 and exon 8 of the *CPQ* gene. By combining the genotype data for these two SNP loci, we identified three different alleles segregating in the three broiler lines. Particular genotypes could be associated with resistance to ascites. We further determined that particular genotypes most associated with resistance overexpress *CPQ* mRNA in three tissues which might explain the role of these alleles in contributing to resistance.

**Conclusions:**

Our findings indicate CPQ is an important determinant of pulmonary hypertension syndrome leading to ascites in broilers. We identified particular SNPs that can be used for marker-assisted selection of broilers for resistance to the disease. Our findings validate WGR as a highly efficient approach to map determinants contributing to complex phenotypic or disease-related traits. The *CPQ* gene has been associated with pulmonary hypertension in genome-wide association studies in humans. Therefore, ascites investigations in broilers are likely to provide insights into some forms of hypertension in humans.

## Introduction

Idiopathic pulmonary arterial hypertension (IPAH), also known as ascites in poultry, is a metabolic disorder attributed to rapid growth in modern broilers. Broilers (meat-type chickens) are selected for rapid growth and increased muscle mass. Since the 1950s, selection has yielded an improved growth rate of about 5% per year [1]. Fast-growing broilers, with enhanced metabolic rate and muscle mass, have higher demands for oxygen. However, the size and capacity of the vital organs, such as the heart and lungs, does not increase proportionately for adequate oxygen delivery in these broilers [1, 2]. The failure of the pulmonary vasculature system to cope with the increasing oxygen requirements leads to constriction of pulmonary arterioles and lack of oxygen in the tissues starting even at embryonic stages [2, 3, 4]. Tissue hypoxia triggers a cascade of events including an increase in vascular pressure in the lungs and pulmonary arteries, right ventricular hypertrophy and valvular insufficiency leading to a drop in cardiac output and hypoxemia [4–8]. This triggers proliferation of red blood cells, which in turn increases the hematocrit value and blood viscosity leading to pulmonary edema, liver damage, accumulation of serous fluid in the abdominal cavity, and right ventricular failure resulting in premature death of the birds [8–11].

Ascites is one of the health traits that is of concern in selective breeding and management practices for broilers. The goals are to minimize economic losses from ascites-induced mortality, reduce hypoxemic affects on meat quality, and improve overall animal well-being [12]. Evaluation of ascites-indicator traits such as cardiac hypertrophy (measured by right ventricular to total ventricular ratios—RV: TV), abdominal fluid, hematocrit value, pulse oximetry has shown to have moderate to high heritabilities [13–15]. For example, heritability of RV: TV ranges from 0.25–0.54, whereas that of abdominal fluid ranges from 0.36–0.44 [13, 15, 16]. This is indicative of the importance of the genetic components that play a key role in ascites [12, 13, 15–21]. Ascites incidence in modern broiler lines can be reduced by effective genetic selection of the birds against susceptibility for the disease. However, because most of the ascites-indicator traits, such as RV-TV ratio and ascitic fluid, can best be measured *post-mortem*, selection of broilers against ascites susceptibility can be difficult. Hence, in current breeding programs, information from siblings and relatives are used for genetic selection of the broiler lines against ascites [7].

Development of genetic markers for selection for resistance to ascites is essential for more effective broiler breeding programs. Although several independent studies have indicated ascites to be a polygenic trait [7, 15, 22–29], little is known about the genes contributing to the disease. We have previously used SNP-based genome-wide association studies to attempt to map determinants for the disease [30–32]. We have now utilized high throughput next-generation sequencing (NGS) for whole-genome resequencing (WGR) to identify regions with multiple SNPs showing association with ascites. In this study, we applied WGR analyses for chromosomes 2 and 9, which identified one of the most promising markers for ascites phenotype. The data identify particular alleles of the carboxypeptidase Q (*CPQ*) gene specifically to conferring resistance to ascites in a gender-biased manner.

## Materials and methods

### Animal ethics statement

All animal procedures were approved by the University of Arkansas Institutional Animal Care and Use Committee under protocol 12039 and 15040, including specific review and approval of the mortality aspects of this research. All birds were observed daily and any moribund birds were immediately euthanized. Ascites does not generally result in the development of outward signs of discomfort, nor does it appear to prevent birds from eating or drinking. Death from ascites is typically the result of the accumulation of fluid in the abdominal cavity which over time exerts pressure on the air sacs and in turn results in congestive heart failure. Daily handling and palpation of the abdominal region could be successful at identifying late stage progression of ascites. However, daily handling and palpation would be stressful on the birds, and would not be effective in identifying birds in early stage progression of ascites. At the end of the experiments all remaining birds were euthanized by either CO_2_ inhalation, or cervical dislocation.

### Bird stocks and selection based on hypobaric chamber trials

The birds used in this study represent one research line (REL) and two commercial elite lines (Lines Y and Z). Birds from the 18^th^ generation of the REL line were used for the WGR data. Parents from 17^th^ generation were mated by artificial insemination of females using pooled semen from males. Eggs were pedigree tracked by hen, immediately wing-banded and randomly assigned to cages in the hypobaric chamber. The chicks belonged to the 18^th^ generation of the REL line. The hypobaric challenge was for six weeks at simulated 2,744 m (9000 ft; 543 mm of Hg) above sea level as described previously [21]. Daily mortalities of the birds were recorded and then necropsied to determine the cause of death and gender of the birds. A bird was classified as ascitic (ascites-susceptible) based on excess fluid accumulation in the abdominal cavity, pericardium and/or hydropericardium [21, 32, 33]. Additional symptoms of ascites included a flacid heart, RVTV ratio and liver lesions [21, 32]. At week six, the surviving birds in the hypobaric chamber were euthanized by cervical dislocation and evaluated for ascites phenotype [21, 32]. Line Y was an elite male commercial line selected over several generations for growth, yield, and efficient feed-conversion rates, while Line Z was a female elite commercial line selected for growth traits as well as reproductive performance. These birds were also challenged in our hypobaric chamber as above.

### DNA isolation from blood samples

At four days of age, 10 μl of blood was extracted via wing vein lancet puncture. DNA was isolated from blood samples using our rapid protocol [34]. Selected DNAs were then further purified by extraction with phenol: chloroform: isoamyl alcohol (50:48:2), and then chloroform: isoamyl alcohol (24:1). Ethanol precipitated DNAs were dissolved in 10 mM TrisCl 0.1 mM EDTA pH 7.5. DNAs were quantified using a Nanovue (GE Healthcare, Pittsburgh, PA).

### DNA preparation for NGS

DNAs submitted for library construction consisted of pools of equal weights of 10 DNAs. Eight pools were generated representing both genders and ascites resistant and susceptibile phenotypes, with two different pools for each gender-phenotype. Pools (100μl) of 40 ng/μl DNA were submitted for bar-coded library construction followed by 2*125 paired-end reads on Illumina HiSeq 2500 to generate at least 65 Gigabases (Gb) per pool. Library construction and sequencing were performed by the Research Technology Support Facility at Michigan State University (East Lansing, MI).

### NGS data analysis and bioinformatics

The NGS sequences (adapters removed) as FASTQ files were aligned to the reference *Gallus_gallus-5* assembly (downloaded from NCBI) in a templated alignment using NGen 14 (Lasergene Suite 14; DNAStar, Madison, WI) using the default parameters. Separate templated alignments were generated for each gender. The templated assembly was then processed using ArrayStar 14 (Lasergene Suite 14) to tabulate SNPs relative to the reference genome and the frequency of each SNP in each DNA pool. The SNP dataset (position, frequency in each pool, Q-score, depth) was exported for individual chromosomes and then imported into Microsoft Excel (Office 2016; Microsoft Corp., Redmond, WA) for further filtering and analysis. SNPs were filtered out with: low depth of coverage (<9) or missing frequencies from one or more replicate pools to reduce false discoveries from low coverage regions. The difference in average SNP frequencies for the two pools for each phenotype was then calculated and was plotted by chromosome position to identify clusters of SNPs with markedly different frequencies between the resistant and susceptible phenotype pools.

### Genome annotations

All genome positions and SNP ID (rs entries) correspond to those from *Gallus_gallus-5* assembly. (Accession ID: GCF_000002315.4) obtained from NCBI.

### Design of PCR, primers, and probes

Specific genomic sequences from the *Gallus_gallus*-5 assembly were manipulated and annotated in SeqBuilder software (DNASTAR Lasergene Suite 14). PCR primers and probes were designed using Primer3 (version 0.4.0; *http*:*//bioinfo*.*ut*.*ee/primer3-0*.*4*.*0/primer3/*) using custom primer size (19-30bp), GC content (40–60%) and Tm range (60–65°C). Primers for reverse transcriptase PCR assays targeted 3’ exons separated by a large intron (>20,000 bp) so as to amplify only cDNAs and eliminate any genomic DNA amplification. Primer and probe sequences were checked for uniqueness by BLAT using the UCSC genome browser. Primers and probes were synthesized by Integrated DNA Technologies (Coralville, IA). Probes incorporated a Zen modification quenched with Iowa Black. Primer and probe sequences designed and used in this study are listed in Table 1.

**Table 1:** Primers, probes, and conditions for RT-qPCR. For each *CPQ* locus: position is the [chromosome]:[chromosomal position] of the 5’ base according to the *Gallus_gallus-5* assembly; Primers are 5’-3’ for forward (F) and reverse (R); Probes are 5’-3’ with allele 1 (P1) labeled with FAM and allele 2 (P2) labeled with HEX. Annealing temperatures for qPCR are indicated in °C.

Assay	Locus	Position (bp)	Primers/Probes^1^	°C
*CPQ* intron 6 SNPs	CPQ_127.7F	2:127670335	CTGCCTAATTGCACTGCCTTTGC	60
	CPQ_127.7R	2:127670591	CATGACTGATTCTGTGGCCTTCCT	
	CPQP1	2:127670526	C**T**ACAATA**A**AAAGAAGAGTTGATTCCC	
	CPQP2		C**C**ACAATA**C**AAAGAAGAGTTGATTC	
*CPQ* exon 8 SNPs	CPQex8F	2:127708143	CGTGATGACCTCAGTAAATACTTCTGG	56
	CPQex8R	2:127708591	CCTGCTGCAAAGGATAAGTTTGCATAC	
	CPQex8P1	2:127708196	TG**C**TTTGGATCCTGAACTGTC	
	CPQex8P2		TG**G**TTTGGATCCTGAACTGTC	
*CPQ* RT-qPCR	CPQ_cDNAex7F	2:127677758	CAATTTCTGGATGAGGGACGGAG	55
	CPQ_cDNAex8R	2:127708283	CAGCATCTCCTCCATGTCAGC	
TBP RT-qPCR	TBPF	3:40848487	GAACCACGTACTACTGCGCT	55
	TBPR	3:40846952	CTGCTGAACTGCTGGTGTGT	

^**1**^ Nucleotides in bold in the probes are the SNPs being assayed.

### 5’-exonuclease allelic discrimination assay (TaqMan genotyping) and DNA sequencing

Targeted SNPs were genotyped by TaqMan 5’-exonuclease assays using qPCR as described previously [31]. Primers and probes for assays are shown in Table 1. Genotype calls were highly repeatable, and were confirmed by product sequencing.

Genotype calls were verified for select samples by purifying the product using RapidTip® (Chiral Technologies; West Chester, PA) and then quantified by Hoechst 33258 fluorescence (Promega Glomax MultiJr Single-Tube Multimode Reader, Thermo Fisher Scientific, Madison, WI). Cleaned samples were submitted for capillary sequencing (Eurofins Genomics, Louisville, KY). Sequence ab1 files were aligned using SeqMan Pro 14 (DNASTAR Lasergene Suite 14) for editing and scoring sequence data.

### Tissue sampling and preparation

Tissue samples were collected from birds with specific allelic patterns at six weeks of age immediately at necropsy. These tissue samples included total heart (both auricles and ventricles), lungs and liver. All freshly harvested specimens for RNA extraction were immediately submerged in of RNAlater solution (Thermo Fisher Scientific, Waltham, MA) at 10:0.5 (v:v) ratio of RNAlater to tissue (thinly sectioned). The suspension was mixed well, equilibrated at 4°C for four to five hours and then stored at -20°C until used. During RNA extraction, the tissues were allowed to thaw before homogenization.

### RNA extraction and gene expression assay

Total RNA from tissue homogenates were extracted using Trizol Reagent (Thermo Fisher Scientific, Waltham, MA) according to the manufacturer’s instructions. RNAs were quantified using a Nanovue (GE Healthcare, Life Sciences, Pittsburgh, PA) and RNA integrity was assessed by agarose gel electrophoresis. Expression levels were determined using single-step RT-qPCR employing Pyrophage® Exo-minus (Lucigen, Middleton, WI). Total RNA (500 ng) was denatured at 65°C for 10 mins, and then added to a mastermix consisting of 1x Pyrophage® 3173 PCR buffer, 1x Evagreen dye (Biotium Inc., Fremont, CA), 0.5 μM dNTPs, 2 μM each of specific forward and reverse primers (Table 1), 0.5 μl of Pyrophage® 3137 DNA polymerase Exo- (Lucigen). The final reaction volume was 20 μl. Negative controls consisted of reactions without RNA. The PCR cycling was initial denaturation at 90°C for 3 mins, 10 cycles of 90°C for 15s, 55°C for 15s, 72°C for 1 min, followed by another 30 cycles of 90°C for 15s, 55°C for 15s, 72°C for 1 min with plate read. All reactions were run in triplicate. TATA-box binding protein (TBP) gene was used as the reference control, and all Ct values were normalized to TBP [35]. Fold change was calculated using the ΔΔCt method.

### Statistical analyses

Genotype and haplotype frequencies were calculated for ascites-resistant and ascites-susceptible phenotypic groups in each line. Expected genotype counts for each phenotype were computed from the genotypic frequency observed for the respective populations. Chi-square values comparing expected to observed counts were then computed using the chitest function in Microsoft Excel to derive P-values and then modified by a simple Bonferroni adjustment based on the number of tests [30]. Genotype data for birds with inconclusive necropsy for ascites were included in the frequency for the entire population but excluded from the phenotypic groups. Two-tailed student t-test with unequal variances was performed to detect differences in gene expression for the alleles. Data has been shown as Mean ± SD. The threshold level of significance used throughout the study was P<0.05.

## Results

### Identification of *CPQ* as a candidate gene from WGR data

**Fig 1:**
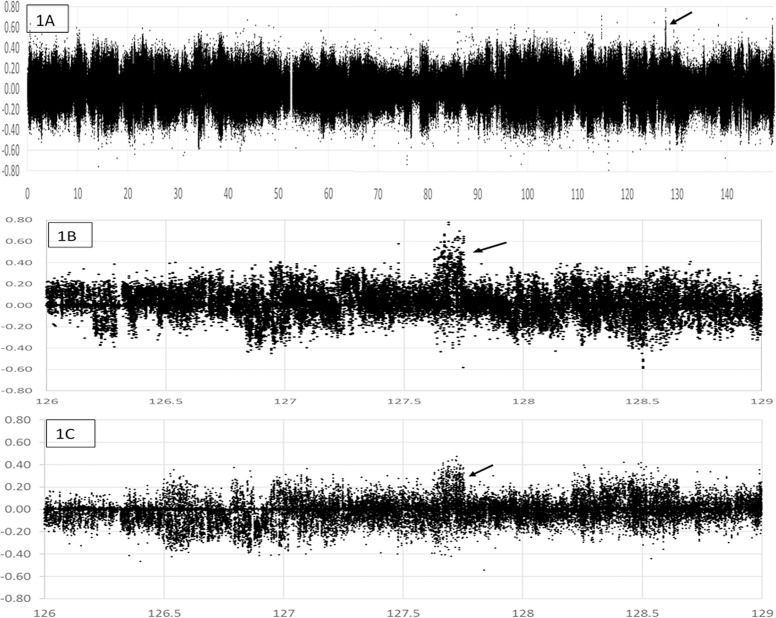
Scatterplot representing GBS data on chromosome 2. Difference in SNP frequencies (Y-axis) is plotted between the average non-reference SNP frequencies of the two replicate pools (resistant less susceptible) relative to chromosome 2 position (X-axis) in Mbp. Positive values indicate that non-reference SNP favors resistance, whereas negative values indicate the SNP favors susceptibility for ascites. **(Panel 1A):** Entire chromosome 2 for males. The black arrow indicates the peak associated with the *CPQ* gene locus. **(Panel 1B):** An expanded view of the peak region ((black arrow) from Panel 1A. **(Panel 1C):** The same region as Panel 1B only for females.

Eight pools of equal DNA quantities were constructed for two replicates for each ascites phenotype (resistant vs. susceptible) and each gender. Illumina libraries were constructed with bar-coding, and paired-end sequenced using Illumina Hiseq 2500 to generate FASTQ 2x125 datasets of ≥65 Gb per DNA pool. The reads were mapped to the *Gallus_gallus-5* reference genome, and SNP data filtered for high-quality SNPs with good read depth (for details see Materials and Methods). The difference in SNP frequencies of the two replicates for each phenotype (resistant minus susceptible) was plotted for each gender by chromosome position to generate scatterplots to identify clusters of SNPs skewed from nearly equal frequencies in both phenotypes. Owing to our earlier interest in chromosomes 2 and 9 [30–32], we first analyzed these chromosomes. The WGR data identified a total of 287,663 SNPs in males and 281,576 SNPs in females for chromosome 9, but we did not find any regions with noticeable skew in the SNP frequencies based on phenotype, even for the two regions we had identified earlier [30, 31]. For Gga2 there were a total of 1,701,018 SNPs in males and 1,700,493 SNPs in females. Comparison of the scatterplots between the males and the females for the chromosome indicated that the differences in the SNP frequencies in females were less for any regions on chromosome 2 as compared to the males. We identified a single region in males from 127.62 to 127.75 Mbps where a cluster of SNPs with high frequency differences of up to about 70% among phenotypes (Fig 1A). We observed the same cluster for females, but the highest difference in SNP frequencies was less 45% suggesting a male-bias for the association (Fig 1B and 1C). In males, the peak spans a 123.1 kbp region, where there were 170 SNPs (post-filtration) with frequency differences ≥ +20% and 77 of these had frequency differences ≥ +40% (S1 Table). Those some-what arbitrary cutoffs were selected based on approximate average variance across the chromosome for each gender. The predominantly positive difference in SNP frequencies indicates that the non-reference SNP cluster is associated with resistance (Fig 1A and 1B). In females, the peak from 127.62 to 127.75 Mbps encompassed a 121.2 Kbp region, where 67 filtered SNPs had a frequency difference of ≥ +20% (S2 Table). We identified five other regions on chromosome 2 in males with less obvious SNP frequency skew (S3 Table). However, none of the other five regions showed as great a difference in SNP frequencies and most showed less significant deviation with respect to phenotype. For each region, we investigated the gene underlying the SNP peak for function and association with ascites-related traits in humans using the NCBI Phenotype-Genotype Integrator (*https*:*//www*.*ncbi*.*nlm*.*nih*.*gov/gap/phegeni*). The peak from 127.62–127.75 Mbp appeared to be associated with the most relevant gene. The peak in males and females encompasses the 3’ end of the gene for carboxypeptidase Q (*CPQ*; also called plasma glutamate carboxypeptidase- PGCP). Most importantly the *CPQ* gene has been associated with hypertension, blood pressure, and heart rate, in human GWAS studies. The peak we observed for this region begins in intron 4 and extends 43 kbp downstream of the final exon, exon 8. In females, we did not observe any additional gene regions that exhibited consistent and significant skew for further analysis.

We, therefore, developed exonuclease assays for further genotyping for this region. Majority of the SNPs within the *CPQ* gene are intronic; five SNPs affect coding sequence including three missense SNPs, and four SNPs are in the 3’UTR of the gene (Table 2). For the exonuclease assays, we chose to target a region with two SNPs in intron 6 which showed one of the highest SNP frequency differences of all the filtered SNPs in males and females, and a missense SNP in exon 8 affecting a conserved lysine residue. The intron 6 SNPs were a T/C SNP at 127,670,527 bp (ID: rs80617053)and a A/C SNP at 127,670,534 bp (ID: rs80617053) (Table 3). From resequencing data, we verified that three other nearby SNPs in intron 6 were in linkage with the targeted SNPs (Table 3). The exon 8 SNP is a G/C SNP (Table 2) at 127708214 bp (ID: rs738850243).

**Table 2:** *CPQ* coding and 3’-UTR SNPs.

Location	Reference ID	Position (bp)	Reference Allele	Variant Allele	Variant Change	Amino acid substitution
Exon 2	rs314744227	127564588	A	G	Non-synonymous	Asn to Ser
Exon 2	rs312495364	127564640	G	A	Synonymous	
Exon 3	rs732164847	127576175	G	A	Non-synonymous	Val to Ile
Exon 4	rs732906703* *	127604368	G	A	Synonymous	
Exon 8*	rs738850243	127708214	G	C	Non-synonymous	Lys to Asn
3’-UTR	rs741020811* *	127708364	A	C		
3’-UTR	rs738375273	127708464	T	A		
3’-UTR	rs735740908	127708512	T	A		
3’-UTR	rs737738647	127708525	A	G		

* Indicates the target SNPs for the exonuclease assay

**Table 3:** Target and linked SNPs for TaqMan assay on intron 6. “Difference in variant allele frequency” columns are calculated by subtracting the average frequency of the non-reference SNP in susceptible from that in resistant from the GBS data.

SNP ID	Position (bp)	Reference Allele	Non-reference Allele	Difference in variant allele frequency- Males	Difference in variant allele frequency- Females
rs80607298	127670474	G	C	+0.50	+0.22
rs80629588	127670479	A	G	+0.43	+0.22
rs80565266	127670525	A	G	+0.55	+0.21
rs80617053*	127670527	T	C	+0.59	+0.23
rs80618855** *	127670534	A	C	+0.51	+0.24

* Indicates the target SNPs for the exonuclease assay

### *CPQ* genotype analysis in three broiler lines

The exonuclease assays were applied to a collection of DNAs from three different broiler lines phenotyped for ascites in hypobaric challenges; the REL line, and two commercial broiler lines, Line Y and Line Z. For the intron-6 SNPs, we genotyped 964 DNAs for REL line birds, 188 DNAs for commercial elite line Y, and 185 DNAs for commercial elite line Z. In the REL line, 66% of all birds and 72% of males homozygous for the non-reference SNP (C/C) were resistant (P = 0.003 and 0.043 respectively; Table 4). Although 62% of the females homozygous for the non-reference SNP were resistant in this line, the difference did not reach the threshold significance of P<0.05. Although the heterozygotes (YM) and the homozygous reference (TA) genotypes statistically deviated from expected both genotypes gave roughly 50:50 resistant and susceptible. The deviation from expected derived from the majority of resistant birds being in the homozygous non-reference genotype. In line Y, 74% of all birds and 81% of males homozygous for the non-reference SNP (C/C) were resistant (P = 0.0005 and 0.0116, Table 4). Homozygous non-reference females were 65% resistant but this did not reach the significance threshold (P = 0.074). It is possible that with more genotype data to increase from the low count (n = 29) that the significance threshold (P<0.05) could be obtained. Unfortunately, we do not have access to additional samples from this line. In Line Z, 82% of all birds and 86% of all males homozygous for the non-reference SNP were resistant (P = 0.0020 and 0.0553; Table 4). Again, the homozygous females for the non-reference SNP did not reach the significance threshold. Therefore, our data indicated that the two intronic SNPs provide a marker for selection for a male-specific resistance to ascites in broiler lines. The homozygous reference genotype for the two SNPs in REL and line Z for both the entire population and for males showed nearly equal (50:50) frequencies for the two phenotypes (REL: P = 0.0006, 0.0063 respectively; Line Z: P = 0.0054, 0.0007 respectively; Table 4). This further implicates the homozygous non-reference genotype for enhanced resistance in the population. However, in Line Y, 64% of the birds in the population and 63% males with the homozygous reference allele were susceptible birds (P = 0.0174, and 0.0302 respectively), indicating that the homozygous reference genotype appears to favor susceptibility in this line. For the most part, the heterozygous genotype approximated 50:50 for both phenotypes in all three lines with the possible exception of females in Line Z. But the counts (n = 12) were low.

**Table 4:** Genotype data for *CPQ* intron-6 SNPs in broiler lines. The REL, Y and Z lines were genotyped using 5’-exonuclease assay where TA is homozygous reference allele, YM (TA/CC) is heterozygous, and CC is homozygous non-reference allele. Genotype frequencies (Freq) were determined for the entire line (All) or for the ascites resistant (R) or susceptible (S) subpopulations based on phenotype in a hypobaric challenge. Count for All in each line include genotypes from birds with missing gender. The samples were also analyzed by gender. P-values for a simple Bonferroni correction (Adj Pval) of chi square test of observed vs. expected are presented.

	Geno-type	All				Male				Female			
Line		Count	R Freq	S Freq	Adj Pval	Count	R Freq	S Freq	Adj Pval	Count	R Freq	S Freq	Adj Pval
**REL**	TA	198	0.46	0.54	0.0006*	87	0.49	0.51	0.0063*	84	0.46	0.54	0.1868
	YM	151	0.50	0.50	0.0641	68	0.56	0.44	0.3296	69	0.45	0.55	0.2625
	CC	615	0.66	0.34	0.0035*	294	0.72	0.28	0.0439*	256	0.62	0.38	0.1538
	TA	86	0.36	0.64	0.0174*	38	0.37	0.63	0.0302*	48	0.35	0.65	0.5745
**Line Y**	YM	36	0.44	0.56	1.2534	17	0.53	0.47	2.2127	19	0.37	0.63	1.4578
	CC	66	0.74	0.26	0.0005*	37	0.81	0.19	0.0116*	29	0.65	0.35	0.0744
	TA	54	0.45	0.55	0.0054*	21	0.38	0.62	0.0007*	31	0.48	0.52	1.5799
**Line Z**	YM	27	0.44	0.56	0.0577	14	0.64	0.36	1.2900	12	0.18	0.82	0.0509
	CC	104	0.82	0.18	0.0017*	71	0.86	0.14	0.0553	32	0.72	0.28	0.1293

* Indicates a significant P value (<0.05).

Similar to that observed for the intron-6 SNPs, analysis of the distribution of the exon 8 (rs738850243) SNP genotypes indicated the homozygous non-reference genotype (C/C) to be significantly associated with the resistant phenotype in all the three lines of REL, Y and Z. Considering all birds with the non-reference genotype (C/C), 69% individuals in REL, 89% in line Y and 90% in line Z were resistant (P = 0.009, 0.00003, 0.0013 respectively; Table 5), implicating the association of the non-reference genotype with resistance in the overall populations. Birds with the homozygous reference genotype (G/G) had about an equal distribution among resistant and susceptible birds at the significance threshold value. In line Y, 60% of the birds with the reference genotype were susceptible while 40% were resistant at P = 0.057 (Table 5). Therefore, the observations for the exon-8 reference genotype distribution were also similar to that of intron-6, where the genotype favored susceptibility only in Line Y. However, for the rs738850243 SNP, we were unable to positively conclude if the non-reference SNP had a gender-specific effect because the ascites-phenotype distribution did not reach the threshold P-value for any of the genotypes for either gender in REL. However, in lines Y and Z, 90% and 93% of the birds in males and 84% and 87% of the birds in females respectively, with homozygous non-reference genotype were resistant at significant threshold (Table 5). We did not observe significant differences between resistant and susceptible birds with the heterozygous genotype in any of the three tested lines.

**Table 5:** Genotype data for *CPQ* exon-8 SNP (NCBI SNP ID: rs738850243) in ascites lines. The REL, Y and Z lines were genotyped using 5’-exonuclease allelic discrimination assay where G is homozygous reference allele, S (G/C) is heterozygous, and C is homozygous non-reference allele. Column and row designations are as described in Table 4.

	Geno-type	All				Male				Female			
Line		Count	R Freq	S Freq	Adj Pval	Count	R Freq	S Freq	Adj Pval	Count	R Freq	S Freq	Adj Pval
**REL**	G	574	0.55	0.45	0.0064*	269	0.59	0.41	0.1116	230	0.52	0.48	0.0931
	S	244	0.65	0.35	0.8667	110	0.70	0.30	0.9183	112	0.60	0.40	2.1702
	C	373	0.69	0.31	0.0092*	167	0.72	0.28	0.2078	164	0.68	0.32	0.0595
	G	103	0.40	0.60	0.0566	45	0.47	0.53	0.2925	58	0.35	0.65	0.3350
**Line** **Y**	S	8	0.37	0.63	1.2975	3	0.33	0.67	1.1090	5	0.40	0.60	2.4799
	C	37	0.89	0.11	0.00003*	20	0.90	0.10	0.0138*	15	0.87	0.13	0.0034*
	G	91	0.50	0.50	0.006*	46	0.52	0.48	0.0052*	41	0.44	0.56	0.5460
**Line** **Z**	S	33	0.73	0.27	1.2233	23	0.87	0.13	0.3764	10	0.40	0.60	1.0935
	C	49	0.90	0.10	0.0013*	30	0.93	0.07	0.0338*	19	0.84	0.16	0.0265*

* Indicates a significant P value (<0.05).

### Assessment of *CPQ* haplotype and genotype patterns in association with pulmonary hypertension

Given that the homozygous non-reference SNPs in intron 6 and exon 8 were individually associated with ascites resistance, we examined the association of the haplotype/genotype patterns formed with these SNPs in the three broiler lines.

Based on the genotype data we constructed haplotypes combining the two intron-6 SNPs (rs80617053 and rs80618855) with the exon-8 SNP (rs738850243). We identified three haplotypes: T-A-G (reference haplotype), C-C-C (non-reference haplotype), and C-C-G (recombinant haplotype), with potentially six different genotypes: genotype G1 (homozygous reference): T-A-G/ T-A-G; genotype G2 (homozygous non-reference): C-C-C/ C-C-C; genotype G3 (homozygous recombinant): C-C-G/ C-C-G; genotype G4 (heterozygous recombinant): C-C-G/ C-C-C; genotype G5 (heterozygous): T-A-G/ C-C-C; and genotype G6 (heterozygous recombinant): T-A-G/ C-C-G. Interestingly, there was no evidence for the “recombinant” T-A-C haplotype in any of the three populations.

As suggested by the association of single SNPs, individuals homozygous for the non-reference G2 consisted of significantly higher frequency of resistant birds as compared to susceptibles in all the three REL, Y and Z lines. This observation was also true considering each gender in all the lines. In REL, 71% of all individuals (P = 0.001), 76% of males (P = 0.051), and 69% (P = 0.027) of females with G2 were resistant(Table 6). In Line Y, 89% of all, 90% of males, and 87% of females with G2 were resistant (P< 0.05; Table 6). In Line Z, 90% of all, 93% of males, and 84% of males with G2 genotype were resistant (P< 0.05; Table 6). We further observed from our haplotype analysis that the reference haplotype (i.e genotype G1) strongly favors susceptibility in the commercial lines Y and Z, considering all birds and for both genders (Table 6). However, in REL, the distribution of resistant and susceptible birds was nearly equal for genotype G1 considering the entire population and males, and slightly higher with 59% susceptibles in females (P = 0.05, Table 6). This further confirms the fact that the homozygous non-reference genotype (G2) favors resistance for ascites in broilers. The reference genotype G1, on the other hand, may favor susceptibility in some of the lines. For the other four genotypes, only G3 and G4 were present at more than 10% but neither showed any association with ascites (Table 6).

**Table 6:** Genotype data combining *CPQ* intron-6 (rs80617053 and rs80618855) and exon-8 (rs738850243) variants for the REL and commercial lines Y and Z. The genotype designations are indicated within brackets for each pattern. Column and row designations are as for Tables 4 and 5. P-values for a simple Bonferroni correction (Adj Pval) of chi square test of observed vs. expected are presented for genotypes with frequency ≥0.10.

Combined Genotypes	All Freq	All R Freq	All S Freq	Adj Pval	Male All Count	Male R Freq	Male S Freq	Male Adj Pval	Female All Count	Female R Freq	Female S Freq	Female Adj Pval
**REL**												
G1	166	0.45	0.55	0.0007*	76	0.51	0.49	0.036*	65	0.41	0.59	0.050
G6	82	0.53	0.47		42	0.55	0.45		32	0.52	0.48	
G5	59	0.51	0.49		20	0.65	0.35		34	0.44	0.56	
G3	178	0.56	0.44	1.040	83	0.63	0.34	2.611	76	0.50	0.50	0.972
G4	94	0.73	0.27	0.061	49	0.73	0.27	0.610	36	0.71	0.29	0.270
G2	287	0.71	0.29	0.001*	137	0.76	0.24	0.051	115	0.69	0.31	0.027*
**Line Y**												
G1	75	0.32	0.68	0.002*	32	0.37	0.63	0.049*	43	0.28	0.72	0.050
G6	4	0.50	0.50		2	0.50	0.50		2	0.50	0.50	
G5	5	0.40	0.60		2	0.00	1.00		3	0.67	0.33	
G3	23	0.61	0.39	1.089	11	0.73	0.27	1.036	12	0.50	0.50	2.141
G4	2	0.50	0.50		1	1.00	0.00		1	0.00	1.00	
G2	35	0.89	0.11	0.00003*	20	0.90	0.10	0.004*	15	0.87	0.13	0.007*
**Line Z**												
G1	45	0.38	0.62	0.0004*	19	0.37	0.63	0.004*	25	0.36	0.64	0.033*
G6	11	0.46	0.54		5	0.40	0.60		5	0.40	0.60	
G5	12	0.50	0.50		8	0.75	0.25		4	0.00	1.00	
G3	32	0.66	0.34	3.865	22	0.68	0.32	1.546	9	0.56	0.44	1.283
G4	18	0.89	0.11	0.141	15	0.93	0.07	0.049*	3	0.67	0.33	0.486
G2	49	0.90	0.10	0.001*	30	0.93	0.07	0.039*	19	0.84	0.16	0.022*

* Indicates a significant P value (<0.05).

### Tissue-specific expression assay

**Fig 2:**
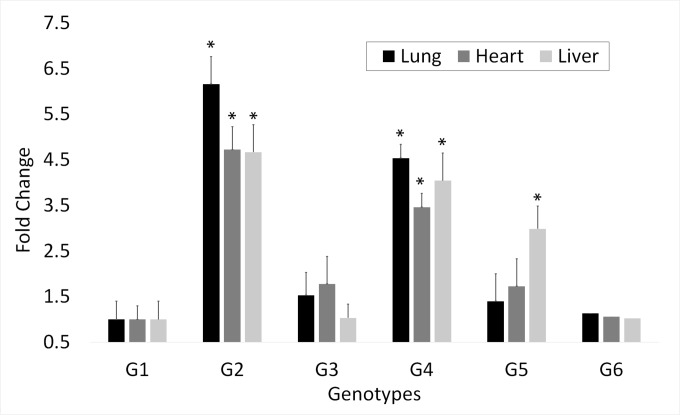
*CPQ* mRNA expression levels for *CPQ* genotypes in lung, heart and liver in REL line broilers. The specific genotypes (G1-G6) are described in the text. Fold change is computed from the mean of the ΔΔCt values with error bars indicating the standard deviation. Sample numbers were: G1, N = 12; G2, N = 14; G3, N = 9; G4, N = 8; G5, N = 11; G6, N = 1. * indicates genotypes significantly different from G1 (homozygous reference haplotype) as determined by two-tailed student’s t-test with unequal variance (P< 0.02).

To further dissect the role of the gene polymorphisms in association with ascites, we assessed the relationship between the various genotypes/haplotypes and *CPQ* gene expression in unchallenged, healthy REL line birds. We focused on expression in heart, lung, and liver, as these are major organs affected in ascites. Our results indicate that the individuals with homozygous non-reference genotype/ haplotype (G2) exhibited the highest level of expression in all the three tissues with a six-fold increase in the lung (P = 0.001), 4.7 folds increase in each of heart (P = 0.001), and liver (P = 0.007) as compared to those with the reference genotype/ haplotype G1 (Fig 2). Individuals with G4 (non-reference intron-6 variants + heterozygous exon-8) had the next highest expression level, consistently in all the three tissues, with fold changes of 4.5 (P = 0.0005), 3.4 (P = 0.0007) and 4.04 (P = 0.018) in the lung, heart, and liver respectively. The fold changes in birds with the genotype/ haplotype G3 (non-reference intron-6 variants + reference exon-8) was much lower followed by that in the heterozygous genotype G5, and then those with the genotype G6 (heterozygous intron-6 variants + reference exon-8) where the expression was the least of all the genotypes/ haplotypes in all the tissues (Fig 2). However, the significance of the expression level did not quite reach the threshold for the latter three genotypes, except for the heterozygous genotype G5 in the liver where the expression was almost three folds higher as compared to G1 (P = 0.025; Fig 2).

## Discussion

The *CPQ* gene encodes a metallopeptidase belonging to the M28 peptidase family. The CPQ protein is thought to play a role in the hydrolysis of circulating peptides in human blood plasma [36], including processing thyroglobulin (Tg) precursor to release thyroxine [37,38]. In human GWAS the *CPQ* gene has shown association with hypertension, blood pressure, heart rate, and electrocardiography. The protein is conserved across many vertebrates and the predicted chicken mature protein is more than 70% identical with those from mouse, rat, monkeys, and human.

Our genotyping data on different broiler lines demonstrate consistent, strong association of the homozygous non-reference genotypes with resistance for both the intron-6 SNP locus (rs80617053 and rs80617055) and exon-8 SNP (rs738850243). The associations are most pronounced in males. Generally, increased ascites resistance is not seen in heterozygotes arguing that the reference allele is dominant over the non-reference allele. The exon 8 SNP would result in a lysine to asparagine substitution at residue 444 of the encoded mature protein. Based on structural data from related proteins in NCBI, the K444N substitution would be on a surface exposed region of the enzyme and not near the active site. Therefore, if this substitution is mediating the QTL effect, it would have to be through affecting some protein-protein interaction, perhaps affecting CPQ activity on a circulating peptide hormone involved in blood pressure homeostasis. Our gene expression data argue that the non-reference genotypes express higher levels of *CPQ* mRNA than the reference genotypes in the three tissues tested; heart, lung, and liver (Fig 2). This implies that one, or more, of the differentially-represented, non-reference SNPs affects regulatory sequences located within or downstream of the 3’ region of the *CPQ* gene; thus impacting relative *CPQ* mRNA expression levels. This could include either transcription factor binding sites or 3’-sequences regulating mRNA half-life (for example: miRNA target sequences).

The identification of the *CPQ* region is based on a collection of SNPs spanning approximately 134 Kbps of Gga2, suggestive of a haplotype block; but our haplotype analysis shows that there is recombination between the intron 6 and exon 8 SNPs. In the REL line, out of 866 DNAs, 354 are with genotypes G3, G4, and G6, indicative of a recombinant allele pairing the non-reference intron 6 SNPs with the reference exon 8 SNP. From the combined genotype data (Table 6) we estimate that this allele represents 30.7% of all alleles with the all reference allele representing 27.3%, and all non-reference allele representing 42% in the REL population. Interestingly we never detect the other recombinant allele pairing the reference SNPs for intron 6 and non-reference SNP for exon 8 in any of the populations. Thus, recombination in the intervening 37,679 bases between the intron 6 and exon 8 SNPs must be extremely rare. Further inspection of the phenotypes associated with G1, G4 and G2 for at least the REL and Line Z indicate that the missense exon 8 SNP cannot be the sole basis for the QTL effect since genotype G4 is heterozygous for the exon 8 SNP (Table 6). In both REL and Line Z, the heterozygotes (G4) for the exon 8 SNP exhibit nearly the same frequency in the resistant birds. In Line Y, there were insufficient numbers of G4 birds for evaluation. Therefore, selection for resistance to ascites should not be based on the exon 8 SNP but is more effective using a combination of SNPs from the tested intronic and exonic loci within this region.

Our genotype-specific expression demonstrates that genotype G2 (homozygous non-reference) has the highest expression in three different tissues (Fig 2). Genotype G4 is expressed at nearly the same level. Genotype G4 is heterozygous recombinant, i.e. it is homozygous for the non-reference SNPs in intron 6 and heterozygous for the missense SNP in exon 8. As genotype G4 is also associated with increased resistance to ascites, this suggests that the level of expression of the mRNA, and perhaps the protein, is critical for contributing to ascites resistance. An increased G4 expression would also be consistent with the intron 6 SNPs being better markers for selection for resistance. Further testing of additional SNPs from this region may elucidate additional genetic diversity (alleles) in this region. Our study indicates that the use of only a single SNP locus may not be as informative as two or more SNP loci for the region. Combining the intron 6 and exon 8 SNPs allowed us to establish that this region shows an association with ascites phenotype even in females, whereas single SNP loci were generally only informative in males.

Prior to this study, we had used multiple 60k SNP panels for GWAS in the REL line, to identify a region on Gga2 around 70.8 Mbp as a candidate for association with ascites [32]. However, that study failed to identify the association of the *CPQ* gene and our new WGR SNP data do not support the region around 70.8 Mbp. There were only three SNPs (rs14250202, rs15154673, and rs14692385) in the 60k SNP panel located within the 134 kbp region. Two of these SNPs showed little to no difference in frequency between phenotypes and the other is monomorphic in the WGR data for the REL samples. Thus, the WGR approach is an unbiased, robust and efficient method to identify genomic regions contributing to complex traits, such as pulmonary hypertension or other diseases. In this context, it is worth noting that neither of the regions we had identified on Gga9 [30, 31] or on GgaZ [32] was confirmed in our WGR analyses. Further preliminary analysis of additional chromosomes from our WGR data has identified an additional 30 regions which appear somewhat similar to the region we found for *CPQ*, where there is a region of skewed SNP frequencies between phenotypes (unpublished). This further validates our WGR (NGS) dataset in identifying QTL regions and reducing false discoveries from our previous GWAS.

Pulmonary hypertension or ascites is a metabolic disorder and is genetically correlated with body weight [12]. Males have higher body weights, and increased oxygen demand, so they are predisposed to ascites. Thus, the effects and manifestation of the disease are more conspicuous in males. This is consistent with the greater SNP frequency skew that we observed in our WGR data for males.

Although CPQ has been associated with hypertension and blood pressure in humans, the precise role of the CPQ protein in mediating these bodily functions are still unclear. Our study indicates that the *CPQ* gene is also associated with pulmonary hypertension in broilers. Similarly, broilers have been shown to form plexiform arteriopathies analogous to those in some forms of human pulmonary hypertension [4]. Our current findings of an association of *CPQ* with hypertension in chickens and the possible contribution of relative expression levels for some alleles may inform biomedical investigations on the role of CPQ in human hypertension. Fast growing broilers with an increased metabolic rate induce an increased secretion of thyroxine, which is deiodinated to triiodothyronine in the liver and kidney [39]. Increased triiodothyronine is positively correlated with increased oxygen consumption. At low-temperature conditions, when increased oxygen is required, the circulating concentration of plasma triiodothyronine increases. The study demonstrated that the plasma thyroxine and triiodothyronine concentrations were significantly higher in healthy birds as compared to ascitic birds. Furthermore, when healthy birds were exposed to cold temperatures, the plasma thyroxin levels were reduced, but eventually recovered except in the ascitic birds [39]. This indicates that the susceptible birds are unable to produce plasma thyroxine at a sufficient rate during increased oxygen demand. There is evidence that CPQ protein plays a role in the release of thyroxine. Therefore, overexpression of the gene (genotypes G2 and G4) could lead to increased thyroxine and triiodothyronine production during oxygen demand and thus contribute to ascites resistance thereby allowing the birds to cope with the higher metabolic rates and increased oxygen requirements.

The *CPQ* gene is the most prominent candidate gene for ascites identified to date, and could be employed in marker assisted selection for increased ascites resistance. The correlation of altered mRNA expression levels for some alleles with resistance to ascites could also relate to the association of CPQ with human hypertension and development of new therapies. Understanding the gene network and downstream protein-protein interactions of the gene is necessary to fully comprehend the importance of the gene in disease-associated phenotypes. Additionally, the WGR approach should be easily adapted to other complex traits and diseases with genetic components in different agricultural species.

## Supporting information

S1 Table Highly Polymorphic SNPs among ascites-resistant and ascites-susceptible male birds forming a peak in and around the *CPQ* gene on chromosome 2. (XLSX)

S2 Table Highly Polymorphic SNPs among ascites-resistant and ascites-susceptible female birds forming a peak in and around the *CPQ* gene on chromosome 2. (XLSX)

S3 Table Regions on chromosome 2 which showed skew for SNP frequency differences from WGR in males. For each region, the table lists the location, the Maximum SNP frequency difference between resistant and susceptible, the calculated P-value (see text), the gene found in the region, and the function of that gene.
(DOCX)
