# Supplementary material for: Whole genome resequencing identifies the CPQ gene as a determinant of ascites syndrome in broilers
Source: PLoS One. 2018 Jan 2;13(1):e0189544. doi: 10.1371/journal.pone.0189544 (PMC5749702; doi:10.1371/journal.pone.0189544)
Supplement: S3 Table — For each region, the table lists the location, the Maximum SNP frequency difference between resistant and susceptible, the calculated P-value (see text), the gene found in the region, and the function of that gene. (DOCX) [file pone.0189544.s003.docx]

**S3 Table. Regions on chromosome 2 which showed skew for SNP frequency differences from WGR in males.** For each region the table lists the location, the Maximum SNP frequency difference between resistant and susceptible, the calculated P-value (see text), the gene found in the region, and the function of that gene.

| Gga 2 Locus (bp) | Maximum SNP frequency difference | P-value | Gene | Function |
| --- | --- | --- | --- | --- |
| 34069064- 34089402 | -0.50 | 0.000327 | *HACL1* | Peroxisomal ɑ-oxidation of 3-methyl branched and 2-hydroxy fatty acids [4]; Refsum Disease [2, 5]. |
| 34105406- 34212779 | -0.59 | 1.265E-34 | *ANKRD28* | Regulation of focal adhesion and cellular migration [6]. |
| 34217438- 34325741 | -0.37 | 1.695E-16 | *LOC101751380* | Unknown. |
| 38501592- 38575922 | +0.51 | 0.0006 | *NEK10* | Serine/threonine protein kinase responsible for regulation of G2/M cell cycle phase transition [7]. |
| 38581179- 38714035 | +0.55 | 0.00021 | *SLC4A7* | Sodium bicarbonate co-transporter [2]. |
| 127544099- 127708625 | +0.66 | 1.43E-29 | *CPQ* | Hydrolysis of circulating peptides in blood plasma [1]; release of thyroxine from thyroglobulin precursor [2, 3]; hypertension, heart rate, blood pressure, electrocardiography [NCBI Phenotype Genotype Integrator]. |

**References**

1. Gingras R, Richard C, El-Alfy M, Morales CR, Potier M, Pshezhetsky AV. Purification, cDNA cloning, and expression of a new human blood plasma glutamate carboxypeptidase homologous to N-acetyl-aspartyl-α-glutamate carboxypeptidase/prostate-specific membrane antigen. Journal of Biological Chemistry. 1999 Apr 23;274(17):11742-50.
2. UniprotKB/SwissProt. http://www.uniprot.org/
3. Ahmetov II, Egorova ES, Gabdrakhmanova LJ, Fedotovskaya ON. Genes and athletic performance: an update. InGenetics and Sports 2016 (Vol. 61, pp. 41-54). Karger Publishers.
4. Foulon V, Sniekers M, Huysmans E, Asselberghs S, Mahieu V, Mannaerts GP, *et al.* Breakdown of 2-hydroxylated straight chain fatty acids via peroxisomal 2-hydroxyphytanoyl-coa lyase a revised pathway for the α-oxidation of straight chain fatty acids. Journal of Biological Chemistry. 2005 Mar 18;280(11):9802-12.
5. Singh I, Pahan K, Singh AK, Barbosa E. Refsum disease: a defect in the alpha-oxidation of phytanic acid in peroxisomes. Journal of Lipid Research. 1993 Oct 1;34(10):1755-64.
6. Kiyokawa E, Matsuda M. Regulation of focal adhesion and cell migration by ANKRD28-DOCK180 interaction. Cell Adhesion & Migration. 2009 Jul 1;3(3):281-4.
7. Moniz LS, Stambolic V. Nek10 mediates G2/M cell cycle arrest and MEK autoactivation in response to UV irradiation. Molecular and Cellular Biology. 2011 Jan 1;31(1):30-42.
